# *In vitro* rescue of genital strains of *Chlamydia trachomatis* from interferon-γ and tryptophan depletion with indole-positive, but not indole-negative *Prevotella spp.*

**DOI:** 10.1186/s12866-016-0903-4

**Published:** 2016-12-03

**Authors:** Noa Ziklo, Wilhelmina M. Huston, Kuong Taing, Mohammad Katouli, Peter Timms

**Affiliations:** 1 University of Sunshine Coast, 90 Sippy Downs Dr, Sippy Downs, Queensland 4556 Australia; 2 University of Technology Sydney, 745 Harris St, Ultimo, New South Wales 2007 Australia; 3 Sexual health and HIV Service, Clinic 87, Sunshine Coast, Queensland Australia

**Keywords:** Tryptophan-synthase, Interferon-γ, Microbiota

## Abstract

**Background:**

The natural course of sexually transmitted infections caused by *Chlamydia trachomatis* varies between individuals. In addition to parasite and host effects, the vaginal microbiota might play a key role in the outcome of *C. trachomatis* infections. Interferon-gamma (IFN-γ), known for its anti-chlamydial properties, activates the expression of indoleamine 2,3-dioxygenase (IDO1) in epithelial cells, an enzyme that catabolizes the amino acid L- tryptophan into N-formylkynurenine, depleting the host cell’s pool of tryptophan. Although *C. trachomatis* is a tryptophan auxotroph, urogenital strains (but not ocular strains) have been shown in vitro to have the ability to produce tryptophan from indole using the tryptophan synthase (*trpBA*) gene. It has been suggested that indole producing bacteria from the vaginal microbiota could influence the outcome of *Chlamydia* infection.

**Results:**

We used two in vitro models (treatment with IFN-γ or direct limitation of tryptophan), to study the effects of direct rescue by the addition of exogenous indole, or by the addition of culture supernatant from indole-positive versus indole-negative *Prevotella* strains, on the growth and infectivity of *C. trachomatis*. We found that only supernatants from the indole-positive strains, *P. intermedia* and *P. nigrescens*, were able to rescue tryptophan-starved *C. trachomatis*. In addition, we analyzed vaginal secretion samples to determine physiological indole concentrations. In spite of the complexity of vaginal secretions, we demonstrated that for some vaginal specimens with higher indole levels, there was a link to higher recovery of the *Chlamydia* under tryptophan-starved conditions, lending preliminary support to the critical role of the IFN-γ-tryptophan-indole axis in vivo.

**Conclusions:**

Our data provide evidence for the ability of both exogenous indole as well as supernatant from indole producing bacteria such as *Prevotella*, to rescue genital *C. trachomatis* from tryptophan starvation. This adds weight to the hypothesis that the vaginal microbiota (particularly from women with lower levels of lactobacilli and higher levels of indole producing anaerobes) may be intrinsically linked to the outcome of chlamydial infections in some women.

**Electronic supplementary material:**

The online version of this article (doi:10.1186/s12866-016-0903-4) contains supplementary material, which is available to authorized users.

## Background

*Chlamydia trachomatis* is an obligate intracellular bacterium with a unique biphasic developmental cycle. The cycle begins with the uptake of the infectious elementary body form (EB) by the host cell. The EB remains in a membrane-bound vacuole termed an inclusion, where it differentiates into the non-infectious, reticulate body form (RB). The RBs undergo cell division. After 8–12 rounds of multiplication, and inclusion growth, RBs asynchronously convert back to the EB form [1, 2]. At 30–68 h post infection (PI), depending on the infecting strain, the EBs are released from the host cell [3]. However, under stressful growth conditions such as nutrient starvation, exposure to antibiotics or immune factors such as interferon-gamma (IFN-γ) [4–6], the chlamydial cycle is disturbed and the RBs convert to enlarged, non-infectious, aberrant bodies (ABs) [1, 3, 7, 8]. Once the stress factor is removed, the *Chlamydia* revert to the active developmental cycle [3, 8, 9].

Genital *C. trachomatis* infections remain a major health problem. Worldwide, an estimated 131 million sexually transmitted *C. trachomatis* infections occur each year [10]. In women, the severity of the infection as well as the probability to progress to complications varies among individuals. Complications such as pelvic inflammatory disease (PID) and infertility are common following *C. trachomatis* infection [11–13] and may be associated with the participant’s inability to fully clear their infection, or a history of repeat infections [13–16]. The proinflammatory cytokine interferon-γ (IFN-γ) is known for its central role in inflammation and autoimmunity [17]. This cytokine is upregulated upon infection [18, 19] and has inhibitory effects on *C. trachomatis* [19, 20]*.* IFN-γ has many effects but for *Chlamydia* most significant appears to be the induction of expression of the enzyme indoleamine 2,3-dioxygenase (IDO), in epithelial cells, that catalyses the degradation of the essential amino acid, L-tryptophan into N-formylkynurenine [21]. Depletion of the host cell tryptophan pools causes the *Chlamydia*, a tryptophan auxotroph, to enter its persistent form [22], evident in vitro by enlarged, aberrant bodies (ABs) (or die at severe depletion) [5, 23]. When the tryptophan is restored, in vitro evidence shows that the *Chlamydia* returns back to its infectious state [24, 25]. While different chlamydial strains have a range of sensitivity levels to IFN-γ treatment in vitro [9, 26], high concentrations are lethal. *C. trachomatis* genital (D-L), but not ocular (A-C) strains, have a functional tryptophan synthase gene (*trpBA*) [25–28], which enables them to synthesise tryptophan from indole. Addition of exogenous indole to the cell culture, can rescue the genital *C. trachomatis* strains from IFN-γ exposure, enabling them to subsequently produce infectious progeny [27, 29, 30].

In addition to the host immune response [16], *C. trachomatis* infection risk is increased during episodes of bacterial vaginosis (BV), which is characterized by reduced levels of lactobacilli and a higher proportion of anaerobic bacteria in the vaginal tract [31–34]. One hypothesis described by Morrison et al. [35], suggested that indole producing bacteria in the vaginal flora might contribute to the survival of the *Chlamydia* by providing a source of indole at the infection site [24, 27, 35–37]. In this study, we directly investigated the effect of indole producing bacteria, such as *Prevotella*, on *C. trachomatis* recovery after tryptophan starvation. Our results show that supernatant from indole producing *Prevotella intermedia* and *Prevotella nigrescens,* but not indole negative *Prevotella bivia*, can rescue *C. trachomatis* after tryptophan starvation *in vitro*. In addition, vaginal secretions from five women had different effects on the recovery of the *Chlamydia* after tryptophan starvation.

## Methods

### *C. trachomatis in vitro* culture conditions

The *C. trachomatis* isolates used in this study included: *C. trachomatis* serotype D (ATCC VR-885), *C. trachomatis* serotype C (ATCC VR-1477). Isolates were routinely cultured in HEp-2 cell line (ATCC CCL-23) with DMEM (Gibco, Australia) containing 5% heat inactivated fetal calf serum (FCS) (Life Technologies, Australia), 120 μg/ml streptomycin (Sigma-Aldrich, Australia), 50 μg/ml Gentamycin (Gibco, Australia), 37 °C, 5% CO_2_. All experiments were conducted in 48-well plates at a multiplicity of infection (MOI) of 0.5. For the IFN-γ-induced tryptophan starvations experiments; 25,000 cells/well were seeded 48 h before infection, in the presence of different concentrations of human IFN-γ (Peprotech, Australia). IFN-γ treatment was replenished every 24 h until the rescue time point. For the tryptophan-depleted media experiments (Jomar Life Research, Australia), 50,000 cells/well were seeded 24 h before infection. At the time of infection, the HEp-2 monolayer was at around 90% confluence. Fresh media and appropriate treatments were supplied to the culture every 24 h and the infectious yields were measured at 36/60/72 h PI (depends on the specific experiment- see figures legend). Infected cells and culture supernatants were then sonicated and used to infect a new HEp-2 cell monolayer in three replicates, for enumeration of recoverable inclusion forming units (IFUs). After staining with anti-HtrA and goat anti rabbit IgG (H + L) Alexa Flour 488 (Invitrogen, Australia), wells were visualized for inclusion presence using fluorescence microscopy (Nikon Eclipse TiS Fluorescent Microscope) [38, 39]. The IFU/ml were determined for each condition by measuring the number of inclusions in multiple wells, taking into account the dilution and volume from the original culture. The limit of detection of the assay is 10^2^ IFU/ml. Rescue experiments using an IFN-γ-induced tryptophan starvation model were conducted following three washes with phosphate-buffered saline (PBS). Rescue experiments using tryptophan-depleted media were conducted with the addition of tryptophan, indole, bacterial isolates supernatant or cervical secretions, in the presence of cycloheximide, at 36 h PI and were incubated for further 36 h. Control cultures with normal tryptophan supply, as well as tryptophan-depleted conditions without rescue, were included in all experiments. In all ‘No rescue’ treatments, cultures were harvested to check chlamydial recovery at 36 h PI. Morphological observation of the chlamydial inclusions in tryptophan-depleted media was made in several of the treatments using *Chlamydia* LPS stain (Cellabs, Australia) and visualised using confocal microscopy (Nikon Eclipse Ti) (Additional file 1: Figure S1). For the morphological observations, cultures were fixed with methanol at 36 h PI (Additional file 1: Figure S1A), and at 72 h PI (Additional file 1: Figure S1B, C).

### *In vitro* rescue of *C. trachomatis* with supernatant from indole positive/negative bacteria

Indole producing bacteria, *P. intermedia* (ATCC 25611) and *P. nigrescens* (ATCC 33563), and a non-indole producing bacterium *P. bivia* (vaginal isolate), were cultured in BHI broth 37 °C/36 h in anaerobic conditions. OD_600_ was measured and corrected for all strains to OD = 1. Indole production was confirmed using Kovac’s reagent (Sigma-Aldrich, Australia). Bacterial broth was centrifuged 3000 × g/10 min/RT and supernatant was collected and filter sterilised with 0.22 μM filter. Supernatant was added to the tryptophan-deprived *C. trachomatis* infected cell culture at 36 h PI. Infected cells and culture supernatants were sonicated at 72 h PI and were used to infect a new HEp-2 cell monolayer for enumeration of recoverable IFUs.

### RNA extraction and reverse transcription

*C. trachomatis* infected cell culture samples were stored in RNA*later*. Total RNA was extracted from the cells using RNeasy mini kit (Qiagen, Australia), according to the manufacturer’s instructions. The RNA concentration and purity was determined using Nano-drop Spectrophotometer. 0.2 μg of total RNA was reverse transcribed using QuantiTect Reverse transcription kit (Qiagen, Australia), in accordance with the manufacturer’s instructions.

### *C. trachomatis* trpBA transcript expression

The primers sequences were taken from Carlson et al., paper [30], with minor changes to complement *C. trachomatis* serotype D. Forward primer for *trpBA* amplification: 5′-GCATTGGAGTCTTCACATGC-3′, and reverse primer: ′3-ACACCTCCTTGAATCAGAGC-5′. Amplification was carried out according to the manufacturer’s instructions using QuntiNova SYBR Green PCR kit (Qiagen, Australia). The cycling program was 95 °C for 2 min followed by 40 cycles of 5 s at 95 °C and 10 s at 60 °C. Transcript levels were quantified using Rotor-GeneQ (Qiagen, Australia). Results were normalized against the mRNA of *C. trachomatis*-specific *ompA* gene transcripts (using previously described primers [40]) in each cDNA preparation. Results are presented as normalised values of 2^-ΔΔCT^.

### Elution of vaginal secretions and Indole concentration measurement

LASIK PVA eye sponges (Visitec) were placed in the posterior fornix of the vagina for two min to absorb secretions [41]. Sponges were immediately placed in −20 °C until vaginal fluid was extracted the same day. Vaginal fluid was eluted from sponges using 300 μl of PBS. Total indoles were quantified using Salkowski’s test [42], modified as described by Szkop et al. [43]. Briefly, serial dilutions of indole (Sigma-Aldrich, Australia) were used in order to generate a standard curve by measuring absorbance at 530 nm, following incubation with Salkowski’s reagent. Indole concentrations were corrected for the dilution factor of the samples.

### Participant details and sample collection procedures

Samples were collected from a small study in reproductive-age women who were either, negative for, or infected with *C. trachomatis,* attending the sexual health clinic in Nambour, Australia. All participants provided informed written consent to participate in the study. Two *Chlamydia* negative and three *Chlamydia* positive women were recruited to the study. *Chlamydia* testing (positive/negative) was performed by the Nambour STI Clinic. High vaginal swab sample and cervical secretion sample were collected from each participant to enumerate chlamydial infection load and indole concentration. Participants’ secretion samples were evaluated for their indole content as described above. Secretions were added to the tryptophan-starved culture at 36 h PI to evaluate the *Chlamydia* recovery effect. The secretions were added in different dilutions; 1:100, 1:1000, 1:10,000, as well as secretions at 1:10,000 dilution with the addition of 0.5 μM indole (Additional file 2: Figure S2).

### Statistical analyses

**Fig. 1:**
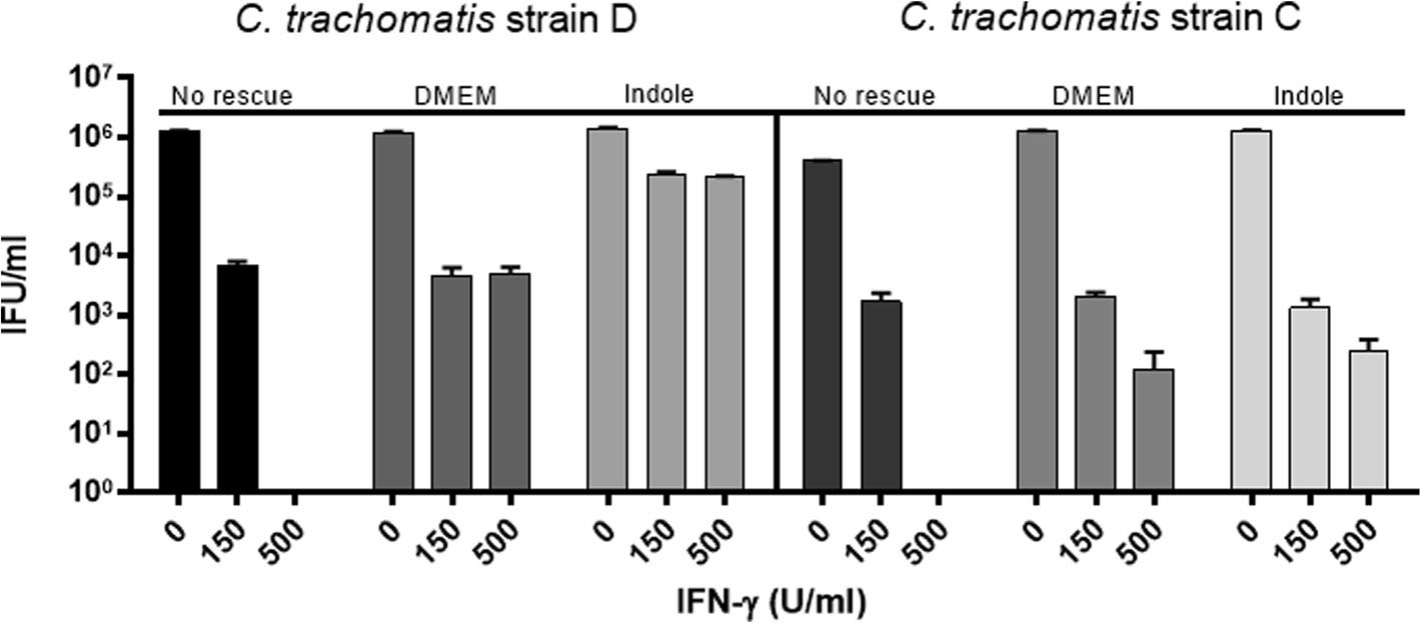
Recovery of IFN-γ treated *C. trachomatis* (ocular; C and genital; D strains), with tryptophan (DMEM) and indole (10 μM). Monolayers of HEp-2 cells were seeded 48 h before infection in the presence of different IFN-γ concentrations (0, 150, 500 U/ml). IFN-γ treatment was replenished every 24 h throughout the whole experiment. Cells were infected with *C. trachomatis* D, at an MOI of 0.5, and were incubated for 36 h. The *Chlamydia* infected cultures were allowed to recover for 24 h in the presence of tryptophan (DMEM) or indole (10 μM). Infected cells and culture supernatants were sonicated and used to infect a new HEp-2 cell monolayer for enumeration of recoverable IFUs. Data are presented as the mean ± SD IFU/ml (*n* = 9) determinations

**Fig. 2:**
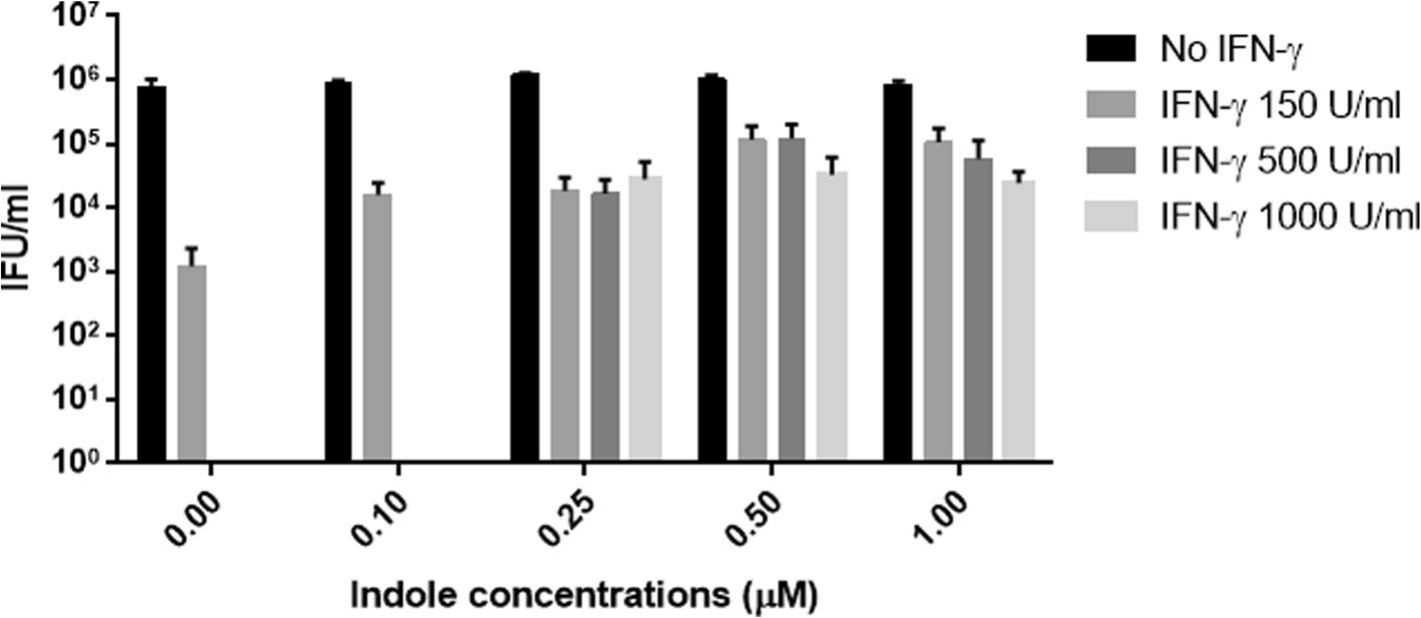
Effect of different indole concentrations on the recovery of *C. trachomatis* D following IFN-γ treatment. Monolayers of HEp-2 cells were seeded 48 h before infection in the presence of different IFN-γ concentrations (0, 150, 500 U/ml). IFN-γ treatment was replenished every 24 h throughout the whole experiment. Cells were infected with *C. trachomatis* D, at an MOI of 0.5, and were incubated for 36 h. The *Chlamydia* infected cultures were allowed to recover for 24 h with different indole concentrations (0.1, 0.25, 0.5, 1 μM). Infected cells and culture supernatants were sonicated and used to infect a new HEp-2 cell monolayer for enumeration of recoverable IFUs. Data are presented as the mean ± SD IFU/ml (*n* = 9) determinations

**Fig. 3:**
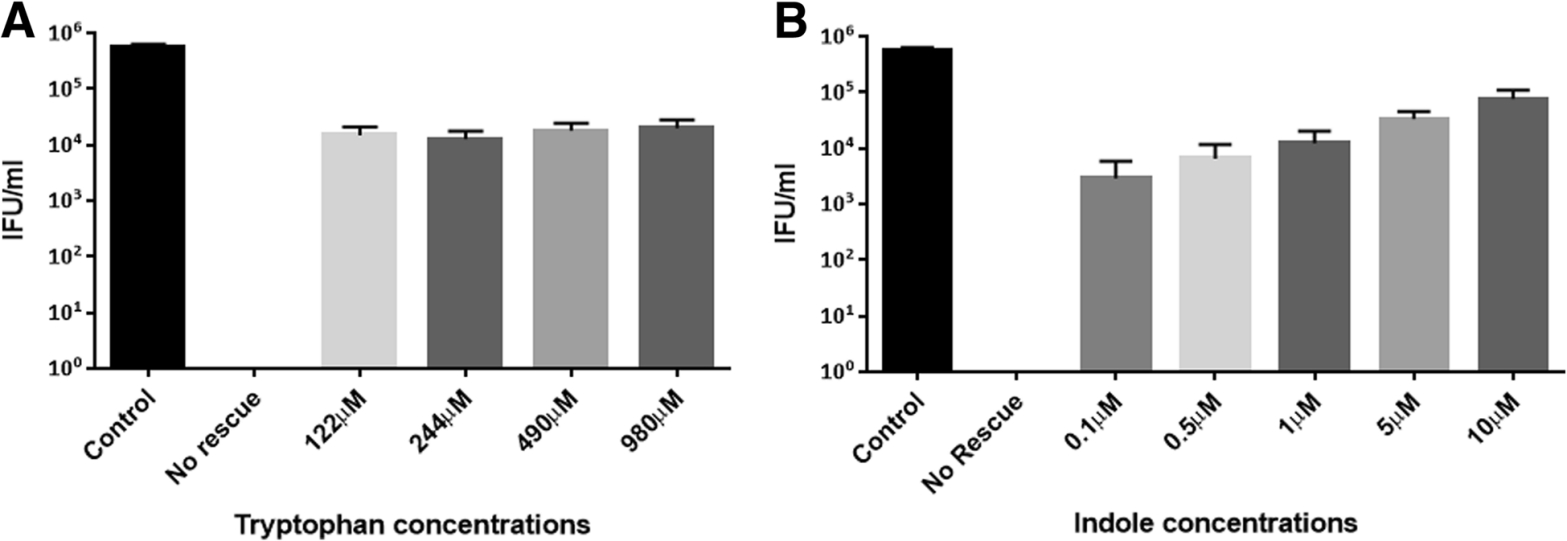
Recovery of tryptophan-starved *C. trachomatis* strain D after rescue with exogenous tryptophan (122–980 μM) or indole (0.1–10 μM). Monolayers of HEp-2 cells were seeded in the presence of tryptophan-depleted media 24 h before infection. Cells were infected with *C. trachomatis* D, at an MOI of 0.5, and were incubated for 36 h. The *Chlamydia* infected cultures were allowed to recover for 36 h with increasing concentrations of tryptophan (**a**) or indole (**b**). Infected cells and culture supernatants were sonicated and used to infect a new HEp-2 cell monolayer for enumeration of recoverable IFUs. Data are presented as the mean ± SD IFU/ml (*n* = 9) determinations

**Fig. 4:**
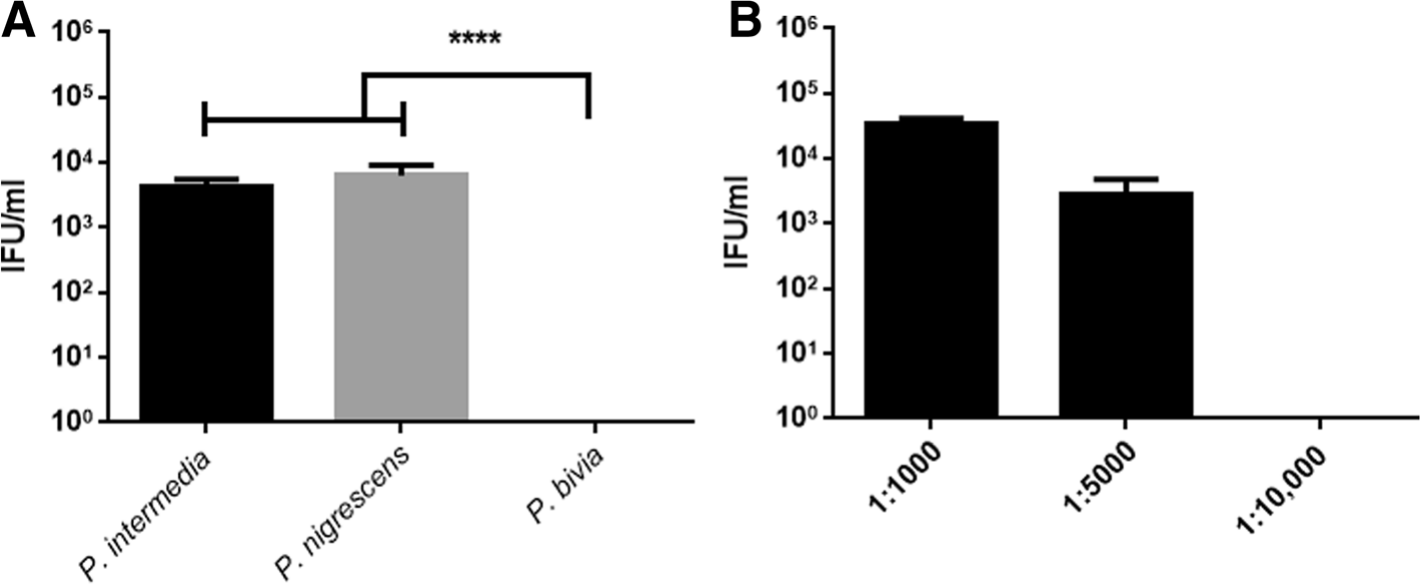
Recovery of tryptophan-starved *C. trachomatis* strain D after rescue with supernatant from indole positive/indole negative bacteria*.* Monolayers of HEp-2 cells were seeded in the presence of tryptophan-depleted media 24 h before infection. Cells were infected with *C. trachomatis* D, at an MOI of 0.5, and were incubated for 36 h. The *Chlamydia* infected cultures were allowed to recover for 36 h in the presence of (**a**) supernatant from indole producing *P. intermedia* and *P. nigrescens*, and a non-indole producer *P. bivia.* Data are presented in dilution of 1:10,000. *P. bivia* is significantly different (*p <* 0.0001). Indole concentrations measured from the growth medium of *P. intermedia* and *P. nigrescens* were 300 μM and 250 μM respectively. **b** A control of the bacterial growth broth (BHI) was added as well. All treatments were added in different dilutions of 1:1000, 1:5000 and 1:10,000 (Additional file 3: Figure S3). Infected cells and culture supernatants were sonicated and used to infect a new HEp-2 cell monolayer for enumeration of recoverable IFUs. Data are presented as the mean ± SD IFU/ml (*n* = 9) determinations. Statistical significance determined via multiple t testing using the Holm-Sidak method, with alpha = 0.05. Each row was analyzed individually, without assuming a consistent SD

**Fig. 5:**
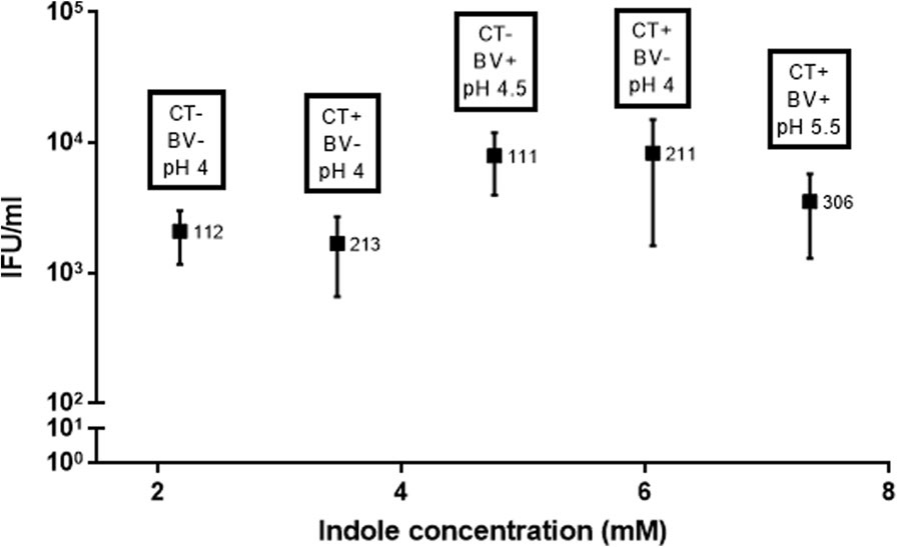
Recovery of tryptophan-starved *C. trachomatis* strain D after rescue with secretions from five (*Chlamydia* positive/negative) participants that have different concentrations of indole in their vaginal secretions. Monolayers of HEp-2 cells were seeded in the presence of tryptophan-depleted media 24 h before infection. Cells were infected with *C. trachomatis* D, at an MOI of 0.5, and were incubated for 36 h. The *Chlamydia* infected cultures were allowed to recover for 36 h in the presence of secretions from two *C. trachomatis* negative participants (111 and 112) and three *C. trachomatis* positive participants (213, 211 and 306). *Chlamydia* test (CT+/CT-), pH and BV conditions are indicated in boxes above each participant. Secretions were added to the cultures at dilution of 1:100. Axis X represent the indole concentrations measured from the participants’ secretions, axis Y represent the recovery effect of the *Chlamydia* in IFU/ml. Infected cells and culture supernatants were sonicated and used to infect a new HEp-2 cell monolayer for enumeration of recoverable IFUs. Data are presented as the mean ± SD IFU/ml (*n* = 9) determinations

**Fig. 6:**
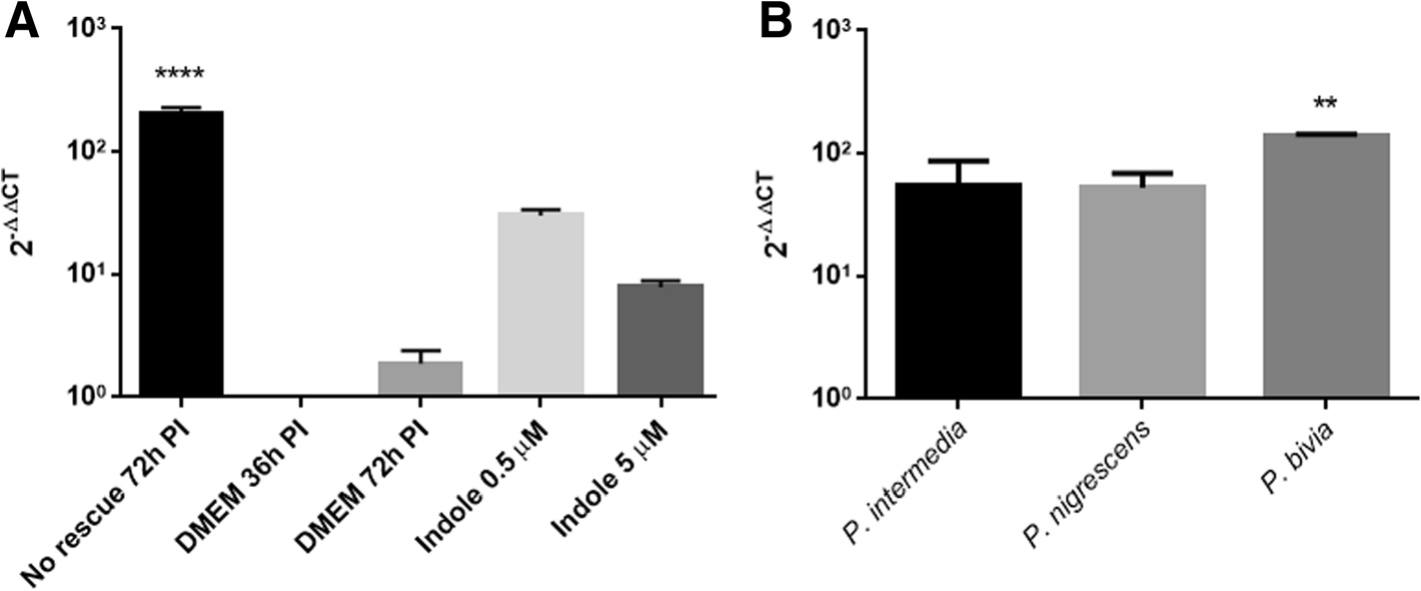
RT-qPCR quantitation of *trpBA* gene mRNA, isolated from HEp-2 cells, infected with *C. trachomatis* strain D during tryptophan starvation conditions and rescue. *C. trachomatis* infected cultures and conditions were as described in the legend of Figs. 4 and 5. **a** Total RNA was isolated from infected cultures grown in tryptophan-depleted media at 72 h PI (‘No rescue’), and after indole rescue in different concentrations (0.5, 5 μM), as well as complete DMEM conditions harvested at 36 and 72 h PI (‘DMEM’). No rescue treatment is significantly different *p* < 0.0001. **b** *trpBA* transcript levels were measured from rescue treatments of infected cultures using indole positive bacterial supernatant (*P. intermedia, P. nigrescens*) and indole negative (*P. bivia*), applied in dilution of 1:10,000. *P. bivia* is significantly different *p* < 0.005

All cell culture experiments (Figs. 1, 2, 3, 4, 5 and 6) were conducted in triplicate. The IFU/ml was determined for each condition by measuring the number of inclusions in multiple wells, and accounting for the dilution and volume from the original culture. Data were analysed using Prism GraphPad V.6 and presented as the mean ± SD IFUs (*n* = 9) determinations. Statistical significance in Fig. 6 was determined using two-way ANOVA and *p*- values were calculated using Tukey’s multiple comparison test. For Fig. 4 statistical significance was determined via multiple t testing using the Holm-Sidak method, with alpha = 0.05, while each row was analyzed individually, without assuming a consistent SD.

## Results

### IFN-γ- induced tryptophan starvation and rescue

We first established an in vitro assay using IFN-γ treated HEp-2 cells, infected with either *C. trachomatis* genital (serovar D) or ocular (serovar C) strains, and demonstrated different abilities of these strains to recover following the addition of tryptophan (fresh ‘DMEM’) or indole (10 μM) (Fig. 1), after IFN-γ treatment. HEp-2 cells were infected with *C. trachomatis* and incubated in the presence of different IFN-γ concentrations (0, 150, 500 U/ml) for 36 h. At the time of recovery; 36 h PI, without rescue (‘No rescue’ treatment), no inclusions were detected. Consistent with the literature, our data showed that both strains were able to recover from IFN-γ treatment (36 h) when the IFN-γ was removed and replaced with fresh DMEM containing 78.3 μM (16 mg/L) L-tryptophan (as per the manufacturer’s description). For *C. trachomatis* D, treatment with 500 units of IFN-γ resulted in no remaining infectious organisms but when fresh DMEM was added, 4.9 × 10^3^ IFUs/ml were recovered. The ocular strain, C, showed 1.2 × 10^2^ IFU/ml recovery under similar conditions. When exogenous indole (rather than DMEM containing exogenous tryptophan) was used for the recovery step, the genital strain D showed a very high (2.1 × 10^5^) recovery of IFUs/ml, as there was no competition from the host cell for indole, whereas the ocular strain C did not show any additional recovery (compared to DMEM alone). Next, we investigated a range of exogenous indole concentrations on recovery and found that levels of 0.25 μM and higher resulted in high levels of recovery, even when the cultures were originally treated with 1000 units/ml of IFN-γ (Fig. 2).

### *C. trachomatis* D recovery using tryptophan or indole in a tryptophan-depleted media model

The model of *C. trachomatis* inhibition using IFN-γ, cultured with DMEM, is problematic, as the media contains large amounts of tryptophan. Therefore, we used a model using tryptophan-depleted media, and added increasing concentrations of tryptophan or indole at 36 h PI to rescue the *C. trachomatis* D from tryptophan starvation (Fig. 3a, b). Exogenous levels of tryptophan were able to effectively rescue the *Chlamydia*, with a maximum level of recovery of 2 × 10^4^ IFU/ml, while there was no competition from the host cell over the tryptophan, as the cultures were treated with cycloheximide (Fig. 3a). Indole rescue on the other hand, showed a maximum recovery level of 7.6 × 10^4^ IFU/ml with concentration dependent increase in the *Chlamydia* infectivity after tryptophan starvation (Fig. 3b). When no rescue treatment was used, there were no inclusions detected at 72 h PI (“No rescue”), as well as at the time of reactivation of the cultures; 36 h PI (data not shown).

### *C. trachomatis* rescue using indole-producing bacterial supernatants

In order to investigate whether supernatant from indole producing bacteria could rescue *Chlamydia*, we chose two indole producing *Prevotella spp.* (*P. intermedia and P. nigrescens*) and one indole-negative strain, *P. bivia*. The bacteria were incubated in brain heart infusion (BHI) medium for 36 h. As the BHI growth medium contains tryptophan, we included the medium alone as a control and tested it at a range of dilutions (1:1000, 1:5000, and 1:10,000). Only supernatant from the indole-positive bacteria (*P. intermedia* and *P. nigrescens*) were able to rescue the *Chlamydia* at the critical dilution of 1:10,000 (Fig. 4) (*p* value < 0.0001 relative to BHI). By comparison, *P. bivia,* which is indole negative, was not able to rescue the *Chlamydia* at the same dilution (*p* value < 0.0001 relative to *P. bivia*). When no rescue treatment was used, there were no inclusions detectable, at 72 h PI, as well as at the time of reactivation of the cultures; 36 h PI (data not shown).

### *C. trachomatis* rescue using vaginal secretions from *Chlamydia* positive and *Chlamydia* negative women

We utilised a combination of in vitro and ex vivo model to test the hypothesis that some of the vaginal indole-producing microbiota may counteract the immune system response mediated by IFN-γ, by providing a source of indole and allows the *Chlamydia* to survive under tryptophan-depleted conditions. Specifically, we tryptophan-starved the *C. trachomatis* D strain using tryptophan-depleted media. We then wanted to determine if vaginal secretions from different women was able to rescue the infectivity of *Chlamydia* to differing degrees, perhaps related to indole levels in these secretions. We therefore evaluated the *Chlamydia* recovery of infectivity compared with the participants’ chlamydial status (positive/negative) and the amount of indole in the vaginal secretions. The indole concentrations from the participants’ secretions (indicated in Fig. 5), ranged from 2.18 to 7.35 mM. Participant’s vaginal secretions were added to the *C. trachomatis* infected culture after tryptophan starvation for 36 h PI. We found higher recovery levels of the *Chlamydia* following rescue with secretions from participants 111 and 211 with relatively high indole concentrations (4.76 mM and 6.06 mM respectively) (Fig. 5). However, using secretions from participant 112 and 213 (who had relatively low indole concentrations in their secretions of 2.18 mM and 3.74 mM respectively), resulted in lower recovery of the *C. trachomatis* after tryptophan starvation in vitro (2 × 10^3^ and 1.6 × 10^3^ IFU/ml respectively). Across all five participants there was no linear correlation of indole and chlamydial recovery, however, four of the five participants were consistent with a trend of higher indole resulting in higher chlamydial infection. Spiking the participants’ secretions with 0.5 μM indole (at dilution of ‘1:10,000 + ’), eliminated some of the differences in the *Chlamydia* recovery that were found between the participants (at dilution of 1:100) (Additional file 3: Figure S3). No significant differences were observed between participant’s secretions treatments in dilutions 1:1000 and 1:10,000 (Additional file 3: Figure S3). When no rescue treatment was used, there were no inclusions detected, at 72 h PI, as well as at the time of reactivation of the cultures; 36 h PI (data not shown).

### trpBA gene expression in tryptophan-starved *C. trachomatis* D following different rescue conditions

In an attempt to gain further support for the hypothesis, that the response of the tryptophan-starved *C. trachomatis* to the availability of tryptophan and indole (added as a rescue treatment 36 h PI) involves their tryptophan biosynthesis genes, we measured the mRNA expression levels of the *trpBA* gene in our indole-producing bacterial supernatant model (Fig. 6). Tryptophan starvation was shown to induce *trpBA* transcription levels in *C. trachomatis* culture (‘No rescue 72 h PI’. Fig. 6a). When exogenous tryptophan is provided via the DMEM medium, *trpBA* levels are switched off. (as expected), and hence any evidence of *trpBA* expression indicates a tryptophan starvation state of the *Chlamydia*. When exogenous indole was provided (at 0.5 and 5 μM), the *trpBA* gene expression levels were again increased to some extent, presumably to convert the indole to more tryptophan. (Fig. 6a). We then assessed the effect of adding bacterial supernatants (at various dilutions) to the chlamydial cultures and found that for *P. bivia* (indole negative) the chlamydial *trpBA* expression was high, confirming that they were tryptophan-starved (Fig. 6b). By comparison, the *trpBA* levels for the two indole positive bacteria (*P. intermedia* and *P. nigrescens*) were significantly lower (*p* value <0.05).

## Discussion

In this study, we investigated the role of indole in the recovery of urogenital *C. trachomatis* infections following tryptophan starvation in vitro. The current hypothesis argues that the availability of indole in the lower genital tract site of women infected with *C. trachomatis*, can influence the level and outcome of the infection. Using both the established IFN-γ model as well as a tryptophan-depleted media model, we found that supernatants from the indole-positive bacteria, *P. intermedia* and *P. nigrescens*, but not indole-negative *P. bivia*, were able to recover *C. trachomatis* D infectivity when added to the cultures at dilution of 1:10,000. Although there is a range of bacterial products being produced by indole-positive bacteria cultured in broth medium, we assume that indole is a critical compound, which directly have a positive effect on *C. trachomatis* recovery after tryptophan starvation *in vitro*. Treatment with supernatant of indole-negative *P. bivia* and the control (BHI) were not sufficient to rescue the *Chlamydia* using the same dilution of 1:10,000. Because the amount of bacteria in a growth medium and the concentration of cytotoxic compounds are much higher than the levels found in vivo, we diluted the bacterial supernatants (1:1000, 1:5000 and 1:10,000). We also included a control of the bacterial growth broth (BHI). We have tested the BHI medium for tryptophan concentration, using commercial tryptophan ELISA kit (ImmuSmol, France), in order to validate our conclusion from this experiment. BHI medium contains 35 μg/ml (0.17 μM) tryptophan and therefore, it might have affected the recovery levels of the tryptophan-starved *Chlamydia* culture. However, when diluting the BHI medium to 1:10,000, the tryptophan in the medium itself was reduced to 3.5 ng/ml, which was previously shown to be insufficient for the recovery of *Chlamydia* after tryptophan starvation [22, 29]. Accordingly, BHI rescue treatment at a dilution of 1:1000 had significantly lower recovery compared to *P. intermedia* at the same dilution (*p* value <0.005, suggesting that indole in the *P. intermedia* supernatant had a beneficial effect, resulting in higher *Chlamydia* recovery in compare to the BHI control (Additional file 3: Figure S3).

Indole concentrations measured from the growth medium of *P. intermedia* and *P. nigrescens* were 300 μM and 250 μM respectively. When diluting the supernatant 1:1000, the indole concentration was decreased to 0.25–0.3 μM (Additional file 2: Figure S2). However, treatment with the indole-positive bacterial supernatant at this dilution, resulted in significantly higher recovery of the *Chlamydia* (*P. intermedia*: 6.8 × 10^4^ IFU/ml, *P. nigrescens*: 3.1 × 10^4^ IFU/ml) (*p* < 0.0001), in comparison to the control in which exogenous indole was directly added to a level of 0.5 μM (3.8 × 10^3^ IFU/ml; Additional file 2: Figure S2). This suggests again that the recovery effect from the bacterial supernatant may be further increased as a result of the tryptophan content in the media, in addition to the indole produced by the indole-positive *Prevotella*.

Measurements of the chlamydial *trpBA* were conducted in order to confirm our recovery data during the different bacterial supernatant rescue treatments. Significantly higher expression levels in *P. bivia* supernatant rescue, suggested that there was no recovery of the tryptophan-starved *Chlamydia* via exogenous indole/tryptophan addition. This confirms our assumption that a non-indole producing bacterium such as *P. bivia*, is not able to rescue the *Chlamydia* after tryptophan starvation. *trpBA* measurements in indole positive bacterial supernatants (Fig. 6b) indicated lower expression levels in comparison to the tryptophan-starved *Chlamydia* (‘No rescue 72 h PI’; Fig. 6a), probably caused by the presence of indole in the media.

In order to investigate whether differences in the indole content in vaginal secretions of women who are negative for, or infected with *C. trachomatis*, have different effects on tryptophan-starved *C. trachomatis* recovery *in vitro*, we used the same tryptophan-depleted media model. We found that low indole content in secretions from participants 112 and 213 corresponded with lower *Chlamydia* recovery indicated by IFU/ml. Higher indole concentration in secretions from participants 111 and 211 corresponded with a higher *Chlamydia* recovery effect at a dilution of 1:100. This might suggest that high indole concentrations in the women’s secretions contribute to higher recovery of the tryptophan-starved *C. trachomatis* culture *in vitro. C. trachomatis*-positive participants 211 and 306 had considerably higher levels of indole in their secretions (7.35 mM and 6.6 mM respectively). This could indicate a link between *Chlamydia* infection status (positive/negative) and the indole concentrations measured from their genital secretions.

Individuals vary in their susceptibility to *C. trachomatis* infections, both new infections, as well as repeat infections [44–46]. While there are many factors that might contribute to this variation, such as individual sexual patterns [46], innate and adaptive immune response [47], the expression and release of the key cytokine IFN-γ [23], one additional factor that might influence this infection variation is the composition of the vaginal microbiome [33, 48, 49]. In most healthy women, lactobacilli are numerically dominant in the lower genital tract, providing protection against a range of pathogenic bacteria and resulting in a lower pH in this environment [50–53]. The replacement of lactobacilli by fastidious anaerobes, such as *Prevotella spp.,* can result in higher pH, dysbiosis and bacterial vaginosis (BV) [48, 54]. It is well known that women with BV have a higher risk of acquiring sexually transmitted infections such *C. trachomatis* [33, 55, 56]. Some of these BV associated *Prevotella* are also indole positive, although this balance may well be quite different between different individuals. Indole production in the lower genital tract can also be associated with higher pH and lower numbers of lactobacilli. Our data clearly show that supernatant from indole positive but not indole negative *Prevotella* strains can rescue *C. trachomatis* from tryptophan starvation, in this in vitro model. It is possible that other indole-producing bacteria (e.g. *Porphyromonas gingivalis, Propionibacterium acnes, Fusobacterium nucleatum, Escherichia coli* or *Enterococcus faecalis*) which have been reported to colonize the genital tract in dysbiosis, could have a similar effect. If this hypothesis is confirmed, it opens up additional means of therapy for women who get frequent *C. trachomatis* infections. Such therapies might include probiotics and other interventions to the vaginal microbiota in order to restore a healthy, acidic, lactobacilli dominant environment.

## Conclusions

Our results give further support to the hypothesis that some members of the genital microbiota, such as *Prevotella*, are able to produce indole and this might influence the natural course of *C. trachomatis* infection in women, by providing a substrate for the *Chlamydia* to produce tryptophan, which enables them to escape the host’s IFN-γ-mediated immune response. We demonstrated that supernatants from indole-producing bacteria were efficient in assisting the recovery of the *Chlamydia* after tryptophan starvation *in vitro*, in comparison to non-indole producers. By directly testing vaginal secretions from a range of women, we found that higher levels of indole in the vaginal secretions from some women contributed to the recovery of tryptophan-starved *C. trachomatis* culture *in vitro*. Thus for the first time we have provided *ex vivo* evidence that indole production in the vagina might have a key role in the outcome of genital *Chlamydia* infections and could lead to the development of novel therapies.

## Additional files

Additional file 1: Figure S1. Microscopic recovery of *C. trachomatis* D following tryptophan starvation and recovery. (DOCX 494 kb)
Additional file 2: Figure S2. Recovery of tryptophan-starved *C. trachomatis* strain D after rescue with secretions from five (*Chlamydia* positive/negative) participants that have different concentrations of indole in their vaginal secretions. (DOCX 61 kb)
Additional file 3: Figure S3. Recovery of tryptophan-starved *C. trachomatis* strain D after rescue with supernatant from indole positive/indole negative bacteria. (DOCX 248 kb)

## Abbreviations

AB: Aberrant bodies
BHI: Brain heart infusion
BV: Bacterial vaginosis
EB: Elementary body
FCS: Fetal calf serum
IDO1: Indoleamine 2,3-dioxygenase
IFN-γ: Interferon-gamma
IFU: Inclusion forming unit
MOI: Multiplicity of infection
PBS: Phosphate-buffered saline
PI: Post infection
PID: Pelvic inflammatory disease
RB: Reticulate body
