# Supplementary material for: In vitro rescue of genital strains of Chlamydia trachomatis from interferon-γ and tryptophan depletion with indole-positive, but not indole-negative Prevotella spp
Source: BMC Microbiol. 2016 Dec 3;16:286. doi: 10.1186/s12866-016-0903-4 (PMC5135834; doi:10.1186/s12866-016-0903-4)
Supplement: Additional file 1: Figure S1. — Microscopic recovery of C. trachomatis D following tryptophan starvation and recovery. (DOCX 494 kb) [file 12866_2016_903_MOESM1_ESM.docx]

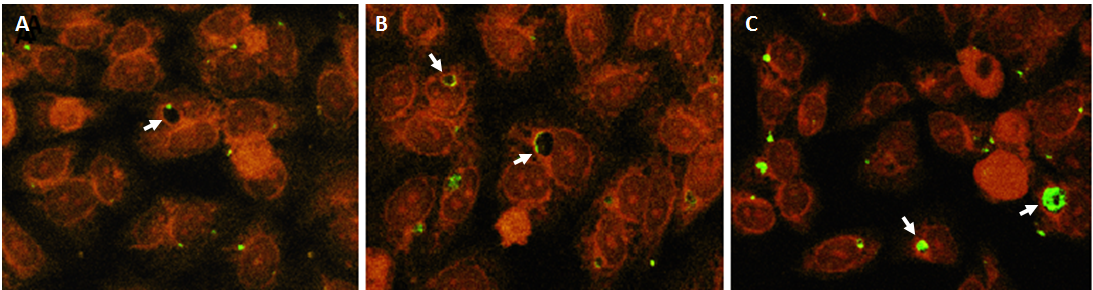


**Figure S1: Microscopic recovery of *C. trachomatis* D following tryptophan starvation and recovery.** Monolayers of HEp-2 cells were seeded in the presence of tryptophan depleted media. Cells were infected with C. trachomatis D, at an MOI of 0.5. Cells and chlamydial inclusions were stained in Chlamydia LPS (Cellabs, Australia). Figure show (A) morphology of chlamydial inclusion in tryptophan-free media 36 h PI. Inclusions are reduced in size and have abnormal morphology. (B) morphology of chlamydial inclusions in tryptophan-free media 72 h PI. Inclusions are reduced in size and have abnormal morphology. (C) morphology of chlamydial inclusions in tryptophan-free media with indole recovery (5µM) added at 36 h PI. Image was taken at 72 h PI. Inclusions have reverted to "normal size" and normal morphology.
