# Supplementary material for: In vitro rescue of genital strains of Chlamydia trachomatis from interferon-γ and tryptophan depletion with indole-positive, but not indole-negative Prevotella spp
Source: BMC Microbiol. 2016 Dec 3;16:286. doi: 10.1186/s12866-016-0903-4 (PMC5135834; doi:10.1186/s12866-016-0903-4)
Supplement: Additional file 2: Figure S2. — Recovery of tryptophan-starved C. trachomatis strain D after rescue with secretions from five (Chlamydia positive/negative) participants that have different concentrations of indole in their vaginal secretions. (DOCX 61 kb) [file 12866_2016_903_MOESM2_ESM.docx]

**Figure S2:** **Recovery of tryptophan-starved *C. trachomatis* strain D after rescue with** **secretions from five (*Chlamydia* positive/negative) participants that have different concentrations of indole in their vaginal secretions.** Monolayers of HEp-2 cells were seeded in the presence of tryptophan depleted media. Cells were infected with *C. trachomatis* D, at an MOI of 0.5, and were incubated for 36 h. The *Chlamydia* infected cultures were allowed to recover for 36 h in the presence of secretions from two *C. trachomatis* negative participants (111 and 112) and three *C. trachomatis* positive participants (213, 211 and 306). Secretions were added at dilutions of 1:100, 1:1000, 1:10,000 and 1:10,000 + (indicates addition of 0.5 µM indole spike to the secretions). Infected cells and culture supernatants were sonicated and used to infect a new HEp-2 cell monolayer for enumeration of recoverable IFUs. Data are presented as the mean ± SD IFU/ml (n=9) determinations.
