# Supplementary material for: In vitro rescue of genital strains of Chlamydia trachomatis from interferon-γ and tryptophan depletion with indole-positive, but not indole-negative Prevotella spp
Source: BMC Microbiol. 2016 Dec 3;16:286. doi: 10.1186/s12866-016-0903-4 (PMC5135834; doi:10.1186/s12866-016-0903-4)
Supplement: Additional file 3: Figure S3. — Recovery of tryptophan-starved C. trachomatis strain D after rescue with supernatant from indole positive/indole negative bacteria. (DOCX 248 kb) [file 12866_2016_903_MOESM3_ESM.docx]

**Figure S3:** **Recovery of tryptophan-starved *C. trachomatis* strain D after rescue with supernatant from indole positive/ indole negative bacteria*.*** Monolayers of HEp-2 cells were seeded in the presence of tryptophan depleted media. Cells were infected with *C. trachomatis* D, at an MOI of 0.5, and were incubated for 36 h. The *Chlamydia* infected cultures were allowed to recover for 36 h in the presence of supernatant from indole producing *P. intermedia* and *P. nigrescens*, and a non-indole producer *P. bivia.* All treatments were added in different dilutions of 1:1000, 1:5000 and 1:10,000. Controls of 'No rescue' at both 36 and 72 h, addition of 'Indole' at 0.5 and 5 µM and the bacterial growth broth (BHI) were added as well. Viability of *C. trachomatis* under normal conditions; 'DMEM' was added at 36 and 72 h PI. Infected cells and culture supernatants were sonicated and used to infect a new HEp-2 cell monolayer for enumeration of recoverable IFUs. Data are presented as the mean ± SD IFU/ml (n=9) determinations.
